# A Cross-Sectional Study of Measles-Specific Antibody Levels in Australian Blood Donors—Implications for Measles Post-Elimination Countries

**DOI:** 10.3390/vaccines12070818

**Published:** 2024-07-22

**Authors:** Kirsten M. Williamson, Helen Faddy, Suellen Nicholson, Vicki Stambos, Veronica Hoad, Michelle Butler, Tambri Housen, Tony Merritt, David N. Durrheim

**Affiliations:** 1 Hunter New England Population Health, Hunter New England Local Health District, Locked Bag 10, Wallsend, NSW 2287, Australia; michelle.butler@health.nsw.gov.au (M.B.); tony.merritt@health.nsw.gov.au (T.M.); david.durrheim@health.nsw.gov.au (D.N.D.); 2 National Centre for Epidemiology and Population Health, Australian National University, 62 Mills Road, Acton, ACT 2601, Australia; tambri.housen@newcastle.edu.au; 3 Australian Red Cross Lifeblood, P.O. Box 354, South Melbourne, VIC 3205, Australia; hfaddy@usc.edu.au (H.F.); vhoad@redcrossblood.org.au (V.H.); 4 School of Health, University of the Sunshine Coast, P.O. Box 200, Petrie, QLD 4502, Australia; 5 Victorian Infectious Diseases Reference Laboratory, Royal Melbourne Hospital, The Peter Doherty Institute for Infection and Immunity, 792 Elizabeth Street, Melbourne, VIC 3000, Australia; suellen.nicholson@vidrl.org.au (S.N.); vicki.stambos@mh.org.au (V.S.); 6 Department of Infectious Diseases, University of Melbourne, Grattan Street, Parkville, VIC 3010, Australia; 7 School of Medicine and Public Health, University of Newcastle, University Drive, Callaghan, NSW 2308, Australia

**Keywords:** measles, antibody, immunity, immunoglobulin, IgG, donor, NHIG

## Abstract

Passive immunisation with normal human immunoglobulin (NHIG) is recommended as post-exposure prophylaxis (PEP) for higher-risk measles contacts where vaccination is contraindicated. However, the concentration of measles-specific antibodies in NHIG depends on antibody levels within pooled donor plasma. There are concerns that measles immunity in the Australian population may be declining over time and that blood donors’ levels will progressively decrease, impacting levels required to produce effective NHIG for measles PEP. A cross-sectional study of Australian plasmapheresis donors was performed using an age-stratified, random sample of recovered serum specimens, collected between October and November 2019 (*n* = 1199). Measles-specific IgG antibodies were quantified by ELISA (Enzygnost anti-measles virus IgG, Siemens), and negative and equivocal specimens (*n* = 149) also underwent plaque reduction neutralisation testing (PRNT). Mean antibody levels (optical density values) progressively decreased from older to younger birth cohorts, from 2.09 [±0.09, 95% CI] to 0.58 [±0.04, 95% CI] in donors born in 1940–1959 and 1990–2001, respectively (*p* < 0.0001). This study shows that mean measles-specific IgG levels are significantly lower in younger Australian donors. While current NHIG selection policies target older donors, as younger birth cohorts become an increasingly larger proportion of contributing donors, measles-specific antibody concentrations of NHIG will progressively reduce. We therefore recommend monitoring measles-specific antibody levels in future donors and NHIG products in Australia and other countries that eliminated measles before the birth of their youngest blood donors.

## 1. Introduction

Australia’s comprehensive national immunisation program and control initiatives have achieved the elimination of endemic measles in Australia [1]. However, measles-specific antibody levels tend to be lower in individuals where immunity is vaccine-derived rather than derived from wild measles virus infection [2]. Declining measles antibody levels have been found in populations where widespread immunisation has led to the interruption of wild-type measles transmission [3,4]. There are concerns that the Australian population may also be experiencing a waning of measles immunity over time.

Normal human immunoglobulin (NHIG) is recommended as post-exposure prophylaxis (PEP) for measles in higher-risk contacts where vaccination is contraindicated [5]. Australian guidelines currently recommend 0.2 mL/kg for infants and pregnant women and 0.5 mL/kg, to a maximum of 15 mL, for immunocompromised people via intramuscular injection [6]. 

NHIG is a sterile solution containing antibodies against various pathogens [7]. Virus-specific antibodies within the preparation bind to the corresponding target, thus preventing or attenuating disease [8]. In Australia, NHIG is produced by CSL Behring through fractionation of human plasma donated to the Australian Red Cross Lifeblood (hereafter “Lifeblood”) [7,9]. Donors must be aged 18 to 75 years or over 75 years if they have donated previously and are medically fit [10]. 

The effectiveness of NHIG in preventing measles is dependent on the concentration of measles-specific antibodies within the preparation [11]. The concentration of measles-specific antibodies within NHIG depends on the antibody levels within the pooled donor plasma from which it is produced [12]. While the minimum effective dose of measles-specific antibodies required (based on contemporary IG products) is unclear, a dose–response effect has been clearly observed [13].

Studies in the US, Germany, New Zealand, Canada, and the UK have found that those born after widespread measles vaccination was introduced, and subsequently living without circulating measles virus, had lower measles-specific antibody levels [14,15]. Corresponding decreases in the measles-specific antibody levels of immunoglobulin products have also been observed [16,17]. Consequently, some countries have increased the dosage and/or changed the route of administration of NHIG, or required that NHIG products achieve a specific measles antibody level [17,18,19,20,21,22]; however, Australian recommendations remain unchanged [6]. Contrastingly, in Australia, NHIG must have a minimum level of hepatitis A virus (HAV) antibodies to be released by CSL Behring. Since 2017, Lifeblood has preferentially directed whole blood-derived plasma from donors aged 60 years and over to CSL Behring for NHIG production, as older donors have been demonstrated to have higher HAV antibody levels [internal data, Lifeblood]. Preferencing donations from older cohorts may affect other specific antibody levels within NHIG.

The objective of this study was to establish if mean measles-specific IgG antibody levels differ between birth cohorts of Australian plasma donors to provide guidance to policymakers. 

## 2. Materials and Methods

### 2.1. Study Population and Design

A cross-sectional study of measles-specific antibody levels in Australian plasmapheresis donors was performed. Subjects had donated at Lifeblood collection centres across Australia between 29 October and 14 November 2019. Recovered serum specimens (residual sera remaining after routine testing) were stratified and randomly selected from the following five age groups, which represented the corresponding birth-year cohorts: (A) 18–29 years old (1990–2001); (B) 30–39 years old (1980–1989); (C) 40–49 years old (1970–1979); (D) 50–59 years old (1960–1969); and (E) 60+ years old (1940–1959).

Demographic data (date of birth, gender, residential postcode, and donation type) were obtained prior to de-identification with a unique study number. Serum (≥1 mL) was aliquoted, and specimens were frozen (−30 °C) and stored before transfer to the Victorian Infectious Diseases Reference Laboratory (VIDRL), Melbourne.

### 2.2. Sample Size Calculation

For estimating the mean antibody level with effect size 0.3 and 90% power, the calculated target sample size per age group was 233 (total target sample size of 1165) [23]. This equated to approximately 10% of plasmapheresis donor specimens received by the Sydney processing centre for specialised testing over one month and approximately 0.25% of the total Australian donor population [24].

### 2.3. Laboratory Testing

All individual specimens were tested using the Enzygnost anti-measles virus IgG diagnostic kit (Siemens Healthcare Diagnostics, Marburg, Germany) on the BEP^®®^ 2000 ELISA processor (Siemens Healthcare Diagnostics) as per manufacturer’s instructions. Measles-specific antibody levels were recorded as optical density (OD) values. The results were classified as positive (OD > 0.2, with OD 0.2–0.4 being ‘low positive’); equivocal (OD 0.1–0.2); or negative (OD < 0.1). Equivocal and negative specimens were retested twice, and the result used was based on concordance with the result interpretation. Previous studies have shown that samples reactive by this ELISA in the range of corrected OD 0.1–0.2 generally have measles-virus neutralising antibody levels of >120 mIU/mL on plaque reduction neutralisation testing (PRNT) [25,26,27,28,29]. Levels ≥120 mIU/mL are considered protective [30,31].

Because PRNT is more sensitive at lower antibody concentrations, all specimens that tested equivocal and negative on ELISA also underwent PRNT [29]. PRNT was performed according to the protocol established for the WHO measles immunogenicity studies of aerosol vaccination project by Cohen et al. [31], based on the original method developed by Albrecht et al. [32]. The 50% neutralising antibody end-point titres (ND_50_) were calculated using the Karber formula, and the results were standardised against the WHO 3rd International Standard (IS) (NIBSC code 97/648) for measles antibodies containing 3000 mIU/mL. The WHO 3rd IS was diluted and tested over a series of ten valid assay runs to determine the geometric mean titre (GMT) and +/− two standard deviations as per recommendation by Cohen et al. [31]. All subsequent assay runs included the diluted WHO 3rd IS for validation purposes and to calculate the sample titres in mIU/mL.

### 2.4. Data Analysis

Statistical analysis was performed with Stata version 14.0. Mean OD values were compared using Student’s *t*-test for variables with two levels (gender, donation type, and donation frequency) and one-way ANOVA with post hoc analysis using Tukey HSD for birth year cohorts. Regarding Student’s *t*-test, the assumption of normality was met by the central limit theorem (i.e., large sample size), and equal variance was observed between groups using a variance ratio test. Although age-group/birth-cohort data appeared skewed, one-way ANOVA was deemed appropriate. Because the ANOVA test is robust, especially as sample size increases and sample sizes for all levels are equal, violations of rules of normality and equal variance that are not extreme can be considered not serious [33]. As per Sullivan (2011), if the largest standard deviation of the groups is less than two times the smallest standard deviation, then the assumption can be considered not violated [34]. 

Kruskal–Wallis test was used to compare median OD values between states/territories due to large differences in sample sizes across the levels. The correlation between OD values and year of birth was determined using the Pearson correlation coefficient. 

Multivariate analysis was performed using a backward, stepwise, linear regression. Variables were initially included, where *p* < 0.25 on parametric tests, and then removed one by one until only significant variables remained. Year of birth was included as a numerical variable, and gender and donation frequency were considered categorical variables. Post-estimation P-P plot, Q-Q plot, and RVF plot were visualised (Appendix A).

## 3. Results

Measles-specific IgG antibody levels were measured in a total of 1200 plasmapheresis donors, with 232 to 245 subjects in each of the five birth year cohorts. One subject was excluded due to discordant results between initial ELISA testing with PRNT and further testing with a second ELISA assay. Therefore, the final study population was *n* = 1199.

The mean OD value was 1.28 (±0.05 95% CI; range 0.00–3.52), well above the assay’s positive cut-off (OD > 0.2). However, there was a consistent, downward trend in the mean measles-specific IgG antibody levels from older to younger donor cohorts. The mean antibody levels were higher in females when adjusted for age. There was no difference in levels based on state/territory of residence or donation type (Table 1).

### 3.1. Measles-Specific IgG Antibody Levels by Subgroup

**Figure 1:**
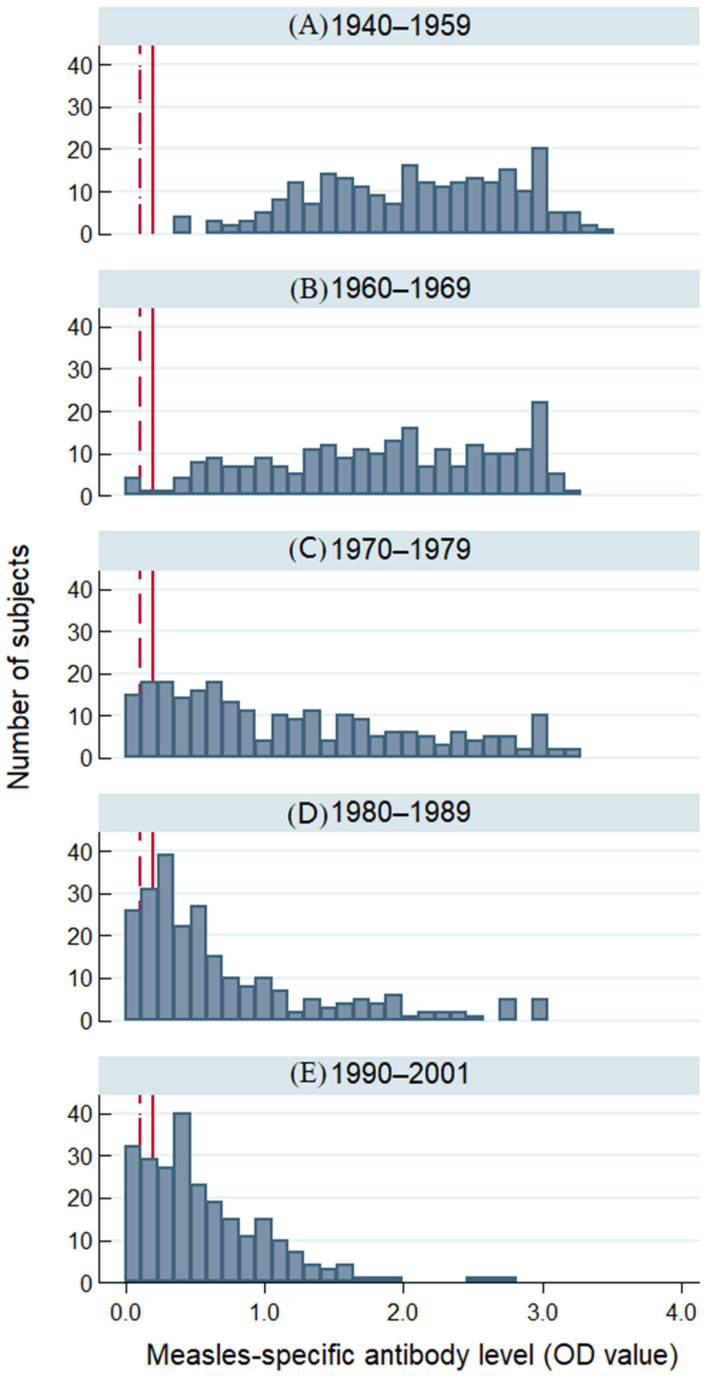
Distribution of measles-specific IgG antibody levels measured by ELISA (optical density values) in Australian plasmapheresis donors that donated from 29 October to 14 November 2019, by birth year cohorts: (**A**) 1940–1959, (**B**) 1960–1969, (**C**) 1970–1979, (**D**) 1980–1989, and (**E**) 1990–2001. The dark red line shows a positive cut-off for assay (OD = 0.2). OD values >0.2 suggest immunity. The dark red dotted line shows an equivocal cut-off (OD = 0.1). OD values 0.1–0.2 are considered equivocal; OD values < 0.1 are negative and therefore considered non-immune.

Although antibody levels were widely dispersed within each age group, there was a clear downward trend in OD values from older to younger cohorts (Figure 1). All 232 donors born before 1960 and 98.7% (236/239) born from 1960 to 1969 had OD values > 0.2, suggesting measles immunity. By comparison, 88.4% (213/241), 81.8% (198/242), and 78.4% (192/245) of donors born in the years 1970–1979, 1980–1989, and 1990–2001, respectively, had OD values > 0.2 (*p* < 0.001).

**Figure 2:**
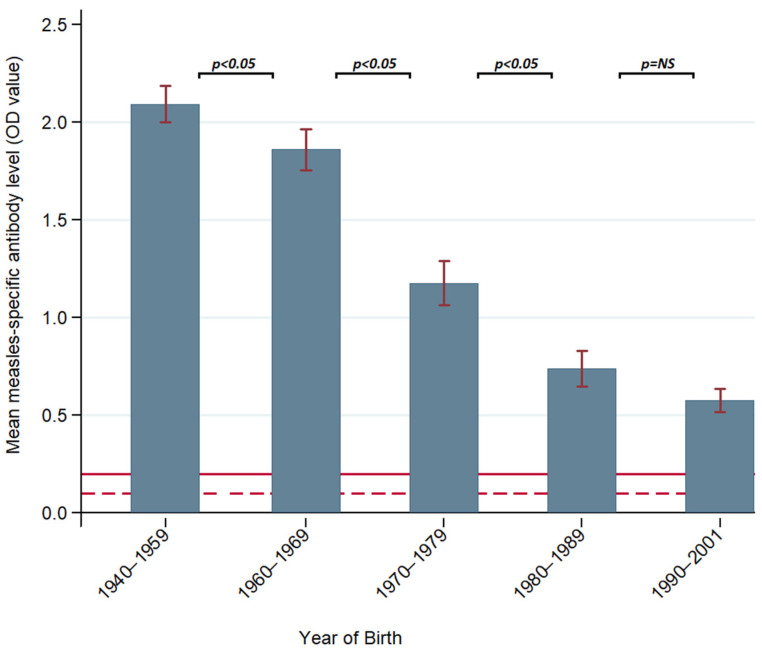
Mean measles-specific IgG antibody optical density (OD) values as measured by ELISA in Australian plasmapheresis donors who donated from 29 October to 14 November 2019, by birth year cohorts. Error bars (vertical) show 95% confidence intervals. Difference bars (horizontal) depict the significance of difference in means between cohorts using one-way ANOVA and Tukey HSD analysis. The dark red line shows a positive cut-off for assay (OD = 0.2). OD values > 0.2 suggest immunity. The dark red dotted line shows an equivocal cut-off (OD = 0.1). OD values 0.1–0.2 are considered equivocal; OD values < 0.1 are negative and therefore considered non-immune.

The mean measles-specific antibody levels decreased significantly from older to younger cohorts (1940–1959: OD 2.09 ± 0.09, vs. 1990–2001: OD 0.58 ± 0.04, 95% CI). Differences between groups were statistically significant in all birth year cohorts (*p* < 0.05) except for the 1980–1989 and 1990–2001 cohorts (Figure 2). There was no significant change to the results when the 1940–1959 cohort (*n* = 232) was separated into two groups (1940–1949 and 1950–1959) nor when subjects in the 1940–1949 group (*n* = 43) were excluded. There was a moderate, negative correlation between measles-specific antibody ELISA OD values and year of birth (Pearson’s r = −0.629, *p* < 0.0001) (Appendix A). 

Univariate analysis did not detect a significant difference in the mean OD values between females (OD 1.32 ± 0.08, 95% CI) and males (1.25 ± 0.07, 95% CI) (*p* = 0.179); however, there was a significant difference when adjusted for age/year of birth (*p* = 0.016) (Appendix A).

There was a significant difference in the mean measles-specific antibody levels between first-time apheresis donors (OD = 1.09 ± 0.08, 95% CI) and repeat donors (OD = 1.39 ± 0.07, 95% CI) (*p* < 0.0001); however, when adjusted for age and gender, the effect of donor status was no longer significant (*p* = 0.524). First-time apheresis donors were generally younger than repeat donors [median age (IQR) 38 years (28–51) vs. 48 years (35–59), respectively]; thus, the relationship between donation frequency and antibody levels was likely confounded by age/year of birth.

Study population proportions by gender and residential state/territory (Table 1) were similar to the broader Australian plasmapheresis donor population (Appendix A). There was no significant difference in the median antibody levels between states and territories (*p* = 0.112).

### 3.2. Multivariate Analysis

Multiple linear regression was performed to predict measles-specific antibody OD value from the year of birth, gender, and first-time vs. repeat donor status. Year of birth and gender added statistically significantly to the prediction (*p* < 0.001 and *p* < 0.01), whilst donor status did not (*p* = 0.981); therefore, donor status was removed from the model. The final model was significant; however, it only explained 40% of the variance in OD values [*F*(2, 1196) = 397.76, *p* < 0.0001, *R*^2^ = 0.399]. It was predicted that as donor age decreased by one year, the OD value decreased by 0.04. Males were predicted to have an OD value of 0.12 less than females of the same age. Post-regression visualisations appeared acceptable for the normality of residuals (P-P and Q-Q plot); the RVF plot was slightly curved, suggesting some heteroscedasticity (Appendix A).

### 3.3. Plaque Reduction Neutralisation Testing (PRNT)

A total of 150 specimens underwent PRNT to determine what proportion of subjects with negative or equivocal ELISA results were still above the assumed correlate of protection. One subject was excluded due to discordant results (see above). This gave a final sample size of *n* = 149, which included all ELISA negative (*n* = 66) and equivocal (*n* = 63) specimens, plus a small random selection of low-positive (*n* = 10) and positive specimens (*n* = 10).

Younger birth cohorts were over-represented amongst the ELISA negative and equivocal specimens: Overall, 75.8% (50/66) and 76.2% (48/63) of negative and equivocal specimens, respectively, came from subjects born after 1979. By contrast, none (0/7) of the negative, equivocal, or low-positive specimens were from subjects born prior to 1960 (Appendix A). Of the 66 specimens that were ELISA-negative, 56.1% (37/66) were above the assumed correlate of protection on PRNT. Of the 63 specimens that were equivocal on ELISA, 96.8% (61/63) were above the assumed correlate of protection. All low-positive (10/10) and positive (10/10) ELISA specimens tested were above the correlate of protection (Table 2).

## 4. Discussion

The effectiveness of NHIG as measles post-exposure prophylaxis is dependent on measles-specific antibody levels in the blood donor population from whom plasma is fractionated to make IG products. This study demonstrated a significant decrease in the mean measles-specific IgG antibody levels from older to younger Australian donor cohorts. The present Lifeblood policy of preferentially using plasma from whole blood donors aged over 60 provides higher antibody levels in current products; however, older birth cohort donors will progressively be replaced by younger ones over decades. In the future, this may result in a decline in measles antibody levels in Australian NHIG and a corresponding reduction in the protection provided to high-risk measles contacts by NHIG, unless measures are taken.

A similar decrease in measles-specific antibody levels was observed across US plasma donor birth cohorts. A sharp decline was noted in donors born after 1963, which coincided with the introduction of measles vaccination in the USA. Levels appeared to plateau in cohorts born in the 1970s and 1980s, followed by another decline after the introduction of a second dose of vaccine in 1989. Another plateau was described in those born after 1990 [14,15].

**Figure 3:**
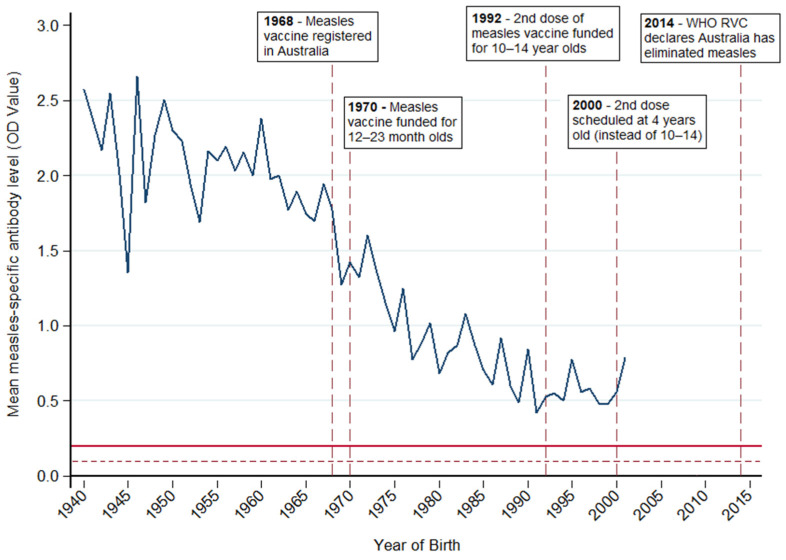
Mean measles-specific IgG antibody levels optical density (OD) values measured by ELISA in Australian plasmapheresis donors who donated from 29 October to 14 November 2019 by year of birth, and milestones for vaccination and control of measles in Australia [35,36]. The dark red line shows a positive cut-off for assay (OD = 0.2). OD values > 0.2 suggest immunity. The dark red dotted line shows an equivocal cut-off (OD = 0.1). OD values 0.1–0.2 are considered equivocal; OD values < 0.1 are negative and therefore considered non-immune.

In our study of Australian plasmapheresis donors, the decline in antibody levels was particularly evident in donors born from the late 1960s onwards (Figure 3). This corresponds with the introduction and subsequent funding of the measles vaccine in Australia in 1968 and 1970, respectively [35]. A steady decrease was observed until the 1990–2001 birth year cohort. A prospective study is required to determine if antibody levels will continue to decline in subsequent birth year cohorts or reach a “steady state” as suggested by the US study.

Australia was verified to have eliminated endemic measles by the World Health Organization in 2014 [36]. However, the absence of endemic transmission in Australia can be demonstrated as early as 2009 and likely occurred even earlier than this [37]. There is an increasing cohort of Australians, born after 2009, who have lived solely in a post-elimination environment and have thus not been exposed to wild-type measles. This cohort is currently too young to be captured by this study; therefore, it would be wise to monitor the measles-specific antibody levels of Australian plasma donor cohorts on a regular basis in the future.

Given the decreasing trend across birth cohorts found by our study, it is anticipated that the mean measles-specific antibody levels of the overall Australian donor population, and thus overall donations, will decrease in the future. The mean antibody level (OD value) for our overall study population was 1.28. It is assumed that donors born after 1970 will form a larger proportion of the total Australian donations in the future. By extrapolating from age-specific levels in our study, the mean antibody level of 2.09 for those 60 years and over in 2019 was estimated to decrease to 1.87 in 2029 and 1.18 in 2039. When adjusted for age, the estimated mean level for total Australian blood donations (plasma and whole blood) received from all age groups in 2019 was 1.30. This was estimated to decrease to 1.01 in 2029 and 0.74 in 2039 (Appendix A). Our estimates suggest that in 10 years’ time, the mean measles-specific antibody levels for donations from those 60 years and over and the annual total Australian donations will be approximately 90% and 80%, respectively, of what they are now. In 20 years, the mean measles-specific antibody levels for donations from individuals 60 years and over, and the total Australian donations, will be approximately 60% of their current level. This is assuming that a “steady state” is maintained in donors born after 1990 and that there is no decay in an individual’s antibody levels over time. 

The mean measles-specific antibody levels were slightly higher in females than males in our study. Nonetheless, both genders showed the same trend towards lower levels in younger birth cohorts. An Australian national rubella serosurvey in 2012–2013 found a lower proportion of rubella seropositive males in the 30–44-year age group, which was thought to reflect the initial vaccination program targeting females only. However, at this time, separate monovalent vaccines were used for rubella and measles, with measles vaccination recommended and funded for both males and females; therefore, it is unlikely to account for the difference in measles immunity between genders [35,38]. However, there is an increasing body of evidence that females typically develop higher antibody responses to a range of vaccines, including the measles–mumps–rubella vaccine [39,40,41]. This has been attributed to hormonal and potentially epigenetic and environmental differences between males and females [40]. However, most vaccine studies do not stratify their data by gender; thus, this area remains under-researched. 

Measles vaccines are part of Australia’s National Immunisation Program and vaccine schedule changes are synchronised across states and territories [35]; thus, similar median antibody levels were expected, although our study was not specifically powered for this. While first-time donors tended to be younger, there was no significant difference in the mean antibody levels between first-time and repeat donors when adjusted for age and gender. The multivariate analysis supported that birth year and gender significantly predicted measles antibody levels, while donation frequency did not. However, the model only explained 40% of the variability in OD values. In future studies, additional variables such as previous measles infection or immunisation would likely improve the model.

Young et al. measured the measles-specific antibody titres in Australian NHIG manufactured between 2010 and 2014 [42], acknowledging that their study was conducted prior to 2017 when Lifeblood began prioritising older donors for NHIG production and therefore titres are expected to have increased. Titres in intramuscular products ranged from 51 to 76 IU/mL when measured by PRNT, which equated to 1.5 to 2.3 times the US Centre for Biologics Evaluation and Research (CBER) standard. Titres did not appear to decrease between products from earlier and later years; however, the study’s time span and sample size were limited. 

If NHIG with a measles antibody concentration of 51 IU/mL was administered using the current Australian guidelines, this would equate to delivery of measles-specific antibodies of at least 10.2 IU/kg for immunocompetent contacts and 25.5 IU/kg for immunocompromised contacts. A study by Endo et al. found that an intramuscular NHIG dose of 10.9 IU/kg given within 5 days prevented clinically evident disease in five out of six (83.3%) paediatric close contacts. Doses of ≥13.2 IU/kg prevented disease in 13 out of 13 (100%) paediatric close contacts [11]. Subsequent modelling by Young et al. estimated that to achieve protection in measles-naïve contacts, an intramuscular dose of 17.5 IU/kg measles-specific antibodies was required [43]. Thus, assuming a measles-specific antibody concentration of NHIG of 51 IU/mL, the current Australian dose of 0.2 mL/kg would under-dose measles-naïve contacts. The decreasing mean antibody levels of Australian blood donor populations forecast by our study and the observed decrease in potency of overseas NHIG products suggest that the measles antibody concentration of Australian NHIG will also fall in the future [16]. 

Multiple international bodies and national studies have recommended an increase in the dose of NHIG as measles PEP [19,20,43,44]. Unlike other countries, routine monitoring of measles-specific antibody concentrations in IG products is not required in Australia [1,22]. The downward trend in antibody levels demonstrated by our study supports the need for monitoring antibody levels in Australian donors and NHIG in the future.

Measures to increase the effectiveness of NHIG include the prioritisation of plasma from older donors, and potential prioritisation from female donors and/or those with a history of measles infection for fractionation. Existing Lifeblood policies mean that, in Australia, male plasma is used solely for direct transfusion, and therefore more female plasma is already directed to plasma for fractionation. Fortunately, because this strategy also works for other diseases for which NHIG-VF is indicated, such as hepatitis A and rubella, this is likely to have enabled Australian products to maintain their efficacy where other countries may not. If antibody levels fall in the future, measles-specific IG products or monoclonal antibodies could be explored, although the manufacture of disease-specific IG precludes the production of other IG from the same source plasma [45]. Revaccination of plasma donors by Modrof et al. showed an increase in measles-specific antibody levels at one month; however, after one year, the levels returned close to baseline [15]. 

This study has limitations. Firstly, due to the sampling approach, first-time apheresis donors were over-represented, and whole blood donors, who are preferentially recruited to provide NHIG, were excluded from the study. As plasma collection volumes increase, the yield of IgG may decrease. As whole blood donors donate less plasma, there may be more IgG per 100 mL. Whilst very frequent plasma donations may cause a decrease in IgG levels [46], the mean apheresis donation frequency in Australia is under four per annum. Additionally, study samples were collected pre-donation. Therefore, it is unlikely this would have significantly altered the findings. Secondly, younger birth cohorts from a post-measles elimination period were too young to be sampled by our study; therefore, we recommend repeating the study regularly in the future. Thirdly, we did not measure antibody levels in NHIG products from the time period. 

PRNT is more sensitive for detecting measles-specific antibodies at lower antibody concentrations; therefore, it was possible that antibody levels below the positive cut-off (OD < 0.2) were underestimated by ELISA. PRNT is the gold standard for estimating measles immunity. However, when compared with ELISA, PRNT is technically demanding, expensive, and difficult to standardise between different laboratories [25,41,47,48]. PRNT detects only functional neutralising antibodies against specific proteins, while ELISA detects antibodies against all viral proteins; additionally, the neutralising antibodies measured by PRNT could belong to all classes of antibodies, while ELISA measures specific classes (IgG and/or IgM and/or IgA) [47].

Previous studies have used ELISA with duplicate testing of a subset of samples using PRNT and observed high agreement of IgG titres between ELISA and PRNT results [25,49], although ELISA values tend to be higher than those of PRNT [47,48]. Somewhat reassuringly in our study, 97% of specimens with “equivocal” ELISA were still above the correlate of protection (120 mIU/mL) on PRNT. This suggests that Australia may have more time before the mean population measles immunity falls below protective levels; however, the overall findings of our study support the need to monitor younger birth cohorts’ immunity in the future. This finding is likely to be shared across countries that achieved interruption of endemic measles transmission before the births of their youngest donors, an effect of delaying concerted efforts to achieve global eradication of measles. Achieving measles eradication would remove the concern about decreasing measles-specific antibody levels.

## 5. Conclusions

Measles-specific antibody levels decreased significantly from older to younger Australian plasmapheresis donors. In the future, when blood donor cohorts targeted for NHIG production progressively include the youngest cohorts, this may impact the measles-specific antibody content of NHIG and jeopardise its effectiveness for passive immunisation against measles. Monitoring measles-specific antibody levels in future blood donor cohorts and NHIG products is recommended in Australia and other countries that eliminated measles before the birth of their youngest donors. 

## Figures and Tables

**Table 1:** Measles-specific IgG antibody levels measured by ELISA (optical density (OD) values) in Australian plasmapheresis donors, 29 October to 14 November 2019, by demography.

	*n* (%)	Mean OD [95% CI]	Standard Deviation	Range	*p*-Value
Total	1199	1.28 [1.23–1.33]		0.00–3.52	
Birth Year Cohort (Age)					
1940–1959 (60+ years)	232 (19.4)	2.09 [2.00–2.18]	0.71	0.36–3.52	**<0.0001**
1960–1969 (50–59 years)	239 (19.9)	1.87 [1.76–1.97]	0.82	0.05–3.17	
1970–1979 (40–49 years)	241 (20.1)	1.18 [1.06–1.29]	0.90	0.00–3.22	
1980–1989 (30–39 years)	242 (20.2)	0.74 [0.65–0.83] ^#^	0.72	0.00–3.00	
1990–2001 (18–29 years)	245 (20.4)	0.58 [0.52–0.64] ^#^	0.47	0.00–2.79	
Gender					
Female	565 (47.1)	1.32 [1.24–1.40]	0.95	0.00–3.52	0.179
Male	634 (52.9)	1.25 [1.17–1.32]	0.95	0.00–3.33	
Donation Type					
Source plasma	1142 (95.3)	1.28 [1.23–1.34]	0.95	0.00–3.52	0.840
Clinical apheresis	57 (4.7)	1.26 [1.01–1.51]	0.96	0.04–3.17	
Donation Frequency					
First-time apheresis donor	440 (36.7)	1.09 [1.01–1.18]	0.90	0.00–3.52	**<0.0001**
Repeat apheresis donor	759 (63.3)	1.39 [1.32–1.46]	0.96	0.00–3.40	
	***n*** **(%)**	**Median OD**	**Interquartile Range**	**Range**	***p*** **-Value**
State/Territory					
Australian Capital Territory	46 (3.8)	1.24	0.54–2.13	0.07–3.00	0.112
New South Wales	275 (22.9)	1.03	0.39–1.98	0.00–3.52	
Northern Territory	3 (0.3)	2.55	1.03–3.00	1.03–3.00	
Queensland	261 (21.8)	1.01	0.45–2.10	0.00–3.33	
South Australia	104 (8.7)	1.44	0.58–2.31	0.09–3.12	
Tasmania	41 (3.4)	1.34	0.61–2.34	0.03–3.00	
Victoria	315 (26.3)	0.98	0.42–1.88	0.00–3.40	
Western Australia	154 (12.8)	1.18	0.37–2.14	0.00–3.15	

^#^ *p* < 0.05 for all birth year cohorts except 1980–1989 and 1990–2001.

**Table 2:** Measles-specific IgG antibody categories measured by ELISA and corresponding measles-neutralising antibody categories by plaque reduction neutralisation testing (PRNT) in a subset of Australian plasmapheresis donors (*n* = 149), 29 October to 14 November 2019.

ELISA Result	PRNT Result		
	Above Correlate of Protection ≥120 mIU/mL *n* (%)	Below Correlate of Protection <120 mIU/mL *n* (%)	Total
Negative	37 (56.1)	29 (43.9)	66
Equivocal	61 (96.8)	2 (3.2)	63
Low-positive	10 (100.0)	0 (0.0%)	10
Positive	10 (100.0)	0 (0.0%)	10
Total	118 (79.2)	31 (20.8)	149

**Figure A1:**
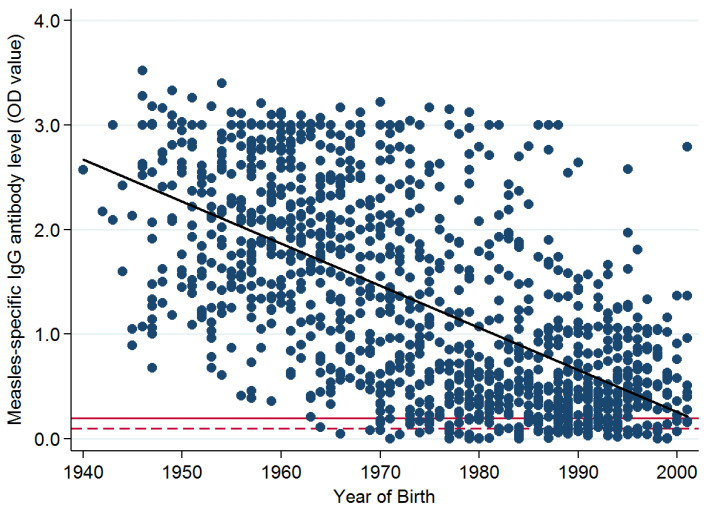
Measles-specific IgG antibody levels (optical density values) measured by ELISA, by year of birth in Australian plasmapheresis donors who donated from 29 October to 14 November 2019. The black line shows the line of best fit. The dark red line shows a positive cut-off for assay (OD = 0.2). OD values > 0.2 suggest immunity. The dark red dotted line shows an equivocal cut-off (OD = 0.1). OD values 0.1–0.2 are considered equivocal; OD values < 0.1 are negative and therefore considered non-immune.

**Figure A2:**
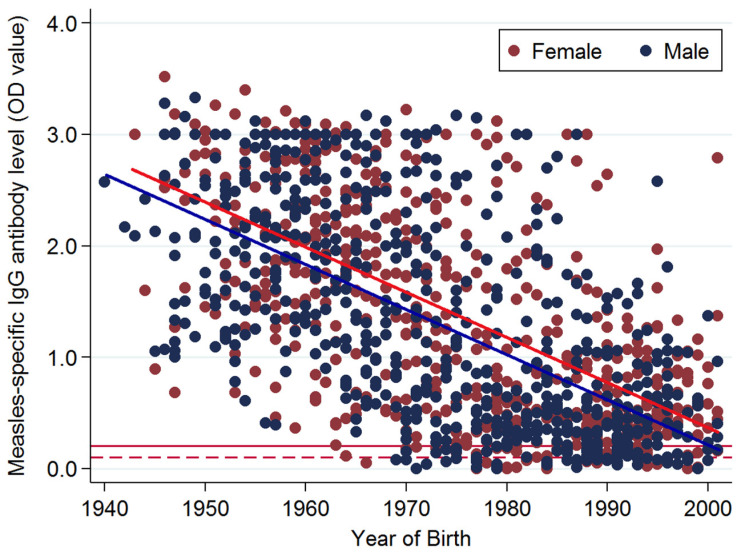
Measles-specific IgG antibody level (optical density values) measured by ELISA by year of birth in Australian plasmapheresis donors who donated from 29 October to 14 November 2019, by gender. Red dots represent female donors and the light red line indicates the line of best fit. Blue dots represent male donors and the blue line indicates the line of best fit. The dark red line shows a positive cut-off for assay (OD = 0.2). OD values > 0.2 suggest immunity. The dark red dotted line shows an equivocal cut-off (OD = 0.1). OD values 0.1–0.2 are considered equivocal; OD values < 0.1 are negative and therefore considered non-immune.

**Figure A3:**
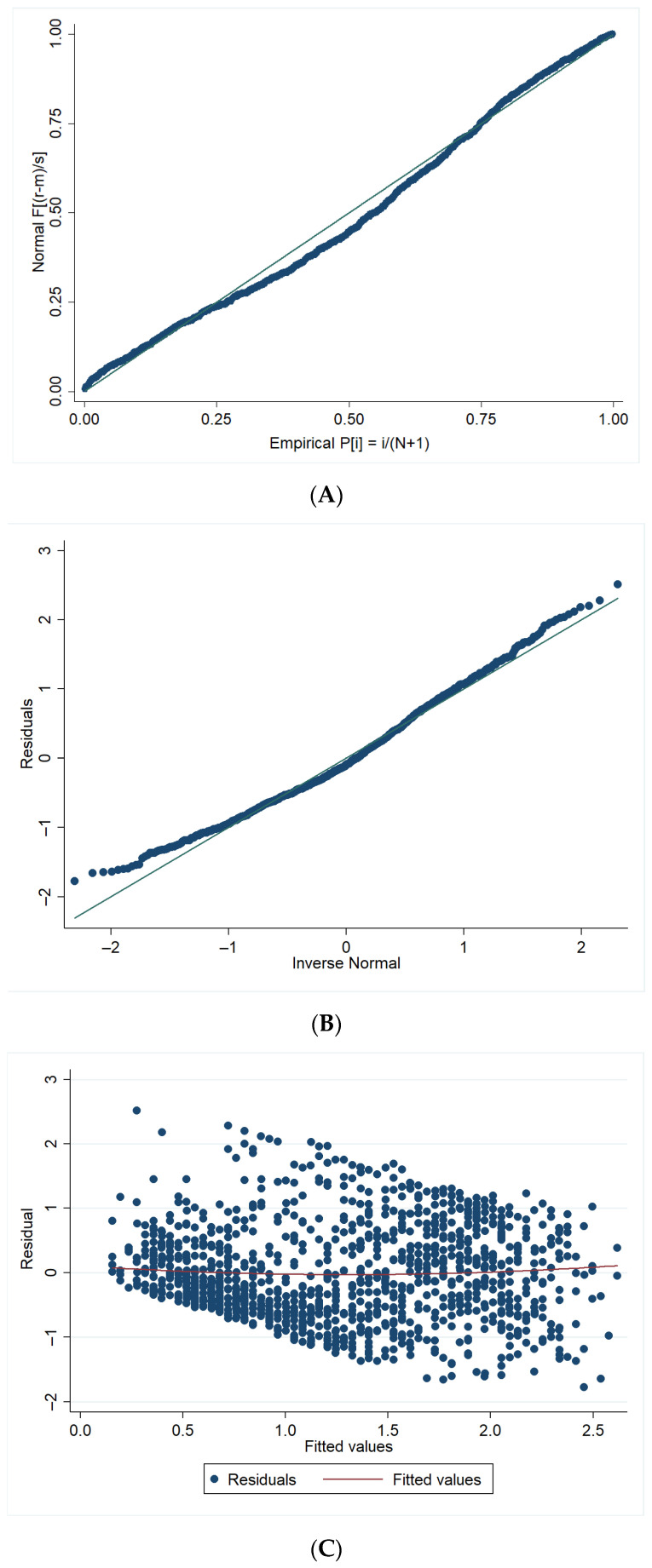
(**A**) P-P plot, (**B**) Q-Q plot, and (**C**) RVF plot of residuals following multiple linear regression analysis of OD value by birth year and gender.

## Data Availability

Data may be made available through the corresponding authors to qualified and interested investigators upon reasonable request.

## Appendix A

**Table A1:** Number and proportion of recovered serum specimens from plasmapheresis donors received by the Australian Red Cross Lifeblood Sydney processing centre in the month of November 2019 [Internal data, Lifeblood].

(A) Age and Gender				(B) State/Territory	
Age Group	Gender	No. by Age & Gender	No. by Age (%)	State/Territory	No. (%)
18–29 years	Female	1954	3565 (28.5%)	ACT	636 (5.1%)
	Male	1611			
30–39 years	Female	1351	2880 (23.0%)	NSW	3237 (25.9%)
	Male	1529		NT	168 (1.3%)
40–49 years	Female	1059	2217 (17.7%)	QLD	2595 (20.8%)
	Male	1158		SA	1014 (8.1%)
50–59 years	Female	900	2022 (16.2%)	TAS	451 (3.6%)
	Male	1122		VIC	3079 (24.6%)
60+ years	Female	749	1820 (14.6%)	WA	1324 (10.6%)
	Male	1071			
All Ages	Female	6013 (48.1%)	12,504 (100%)	All States/Territories	12,504 (100%)
	Male	6491 (51.9%)			

**Table A2:** Demographics of a subset of study population (*n* = 149) that underwent measles-specific IgG antibody testing by ELISA and measles neutralising antibody testing by PRNT.

	All Study Subjects	Subset of Subjects Who Underwent PRNT				
	*n* (%)	Total *n* (%)	ELISA Negative *n* (%)	ELISA Equivocal *n* (%)	ELISA Low Positive *n* (%)	ELISA Positive *n* (%)
Total	1199	149 (100)	66 (100)	63 (100)	10 (100)	10 (100)
Birth Year Cohort (Age)						
1940–1959 (60+ years)	232 (19.4)	7 (4.7)	0 (0.0)	0 (0.0)	0 (0.0)	7 (70.0)
1960–1969 (50–59 years)	239 (19.9)	6 (4.0)	2 (3.0)	1 (1.6)	0 (0.0)	3 (30.0)
1970–1979 (40–49 years)	241 (20.1)	30 (20.1)	14 (21.2)	14 (22.2)	2 (20.0)	0 (0.0)
1980–1989 (30–39 years)	242 (20.2)	49 (32.9)	20 (30.3)	24 (38.1)	5 (50.0)	0 (0.0)
1990–2001 (18–29 years)	245 (20.4)	57 (38.3)	30 (45.5)	24 (38.1)	3 (30.0)	0 (0.0)
Gender						
Female	565 (47.1)	55 (36.9)	17 (25.8)	30 (47.6)	3 (30.0)	5 (50.0)
Male	634 (52.9)	94 (63.1)	49 (74.2)	33 (52.4)	7 (70.0)	5 (50.0)

## Appendix B. Estimation of Levels in Current and Future Australian Donor Populations

Age-group-specific mean OD values obtained from this study were applied to the total Australian blood donation (whole blood and plasma) demographics for the year 2019 [internal data, Lifeblood], to estimate an age-adjusted mean antibody level for the total Australian blood donations in 2019 (see Appendix B Table A3). Estimation of the mean measles-specific antibody levels for future total Australian blood donations (whole blood plus plasma) in the years 2029 and 2039 were made using the following assumptions:

1. The distribution of whole blood and plasma donations across age groups in the future will be similar to that of the Australian donations in 2019.
2. The current age groups in this study and their corresponding age-specific mean antibody level will become the next consecutive age group and antibody level in ten years’ time; that is, the current 30–39-year age group in 2019 with mean antibody level = X will become the 40–49-year age group in 2029 whose mean antibody level = X, and the 50–59-year age group in 2039 whose mean antibody level = X.
3. There is no decay of antibody levels within donors as they age; that is, the mean antibody level = X for 30–39-year-olds in 2019 will become the mean antibody level = X for 40–49-year-olds in 2029 without any waning.
4. As the antibody levels of youngest donors (18–29-year-olds) in future cohorts cannot be predicted, the mean antibody level for 18–29-year-olds in 2019 is used for 18–29-year-olds in 2029 and 2039, acknowledging that this may be an overestimate.

**Table A3:** Total Australian blood donations (whole blood and plasma) by age group in 2019 [internal data, Lifeblood]. Age-specific mean measles IgG antibody levels in Australian blood donors in 2019 based on study results; estimated age-adjusted mean antibody levels for the total Australian blood donations in 2019; predicted age-adjusted mean antibody levels for total Australian blood donations in 2029 and 2039.

Age Group (Years)	Number of Total Blood Donations (Plasma + Whole Blood) in 2019	%	Mean Measles-Specific IgG Antibody Levels in 2019 [95% CI]	Estimated Mean Antibody Levels in 2029 *	Estimated Mean Antibody Levels in 2039 *
18–29	327,130	22.14%	0.58 [0.52–0.64]	0.58	0.58
30–39	278,054	18.81%	0.74 [0.65–0.83]	0.58	0.58
40–49	258,875	17.52%	1.18 [1.06–1.29]	0.74	0.58
50–59	291,846	19.75%	1.87 [1.76–1.97]	1.18	0.74
60+	321,959	21.79%	2.09 [2.00–2.18]	1.87	1.18
Total	1,477,864	100.00%			
Age-adjusted mean antibody level for annual total Australian blood donations			1.30	1.01	0.74

* Assumed that the current age groups in the study and their mean antibody level will become the next consecutive age group in 10 years (e.g., current 18–29-year-olds with a mean OD value of 0.58 will become the 30–39-year age group in 2029). Assumed antibody levels plateau in donors born after 1990 (which is unconfirmed); therefore, projected age-adjusted levels for 2029 and 2039 may be overestimated.
